# Association between pancreatic cancer and diabetes: insights from a retrospective cohort study

**DOI:** 10.1186/s12885-023-11344-w

**Published:** 2023-09-11

**Authors:** Fakhrddine Amri, Chifaa Belkhayat, Asmae yeznasni, Hajar Koulali, Rachid Jabi, Abdelkrim Zazour, Naima Abda, Mohammed Bouziane, Zahi Ismaili, Ghizlane Kharrasse

**Affiliations:** 1 Department of Hepato-Gastroenterology, Mohammed VI University Hospital, Oujda University, Oujda, BP 4806, 60049 Morocco; 2 Department of General Surgery, Mohammed VI University Hospital, Oujda, Morocco; 3 Digestive Diseases Research Laboratory (DSRL), Faculty of Medicine and Pharmacy, Mohammed First University, Oujda, Morocco; 4 Laboratory of Epidemiology, Clinical Research, and Public Health, Faculty of Medicine and Pharmacy, Mohammed First University, Oujda, Morocco

**Keywords:** Pancreatic adenocarcinoma, Diabetes mellitus, Poor prognosis, Risk factors

## Abstract

**Background:**

Studies investigating the prevalence of pancreatic cancer have revealed a heightened risk of 1.5 to 2.0 times among individuals with long-standing type 2 diabetes mellitus.

**Aims:**

We aimed to estimate the prevalence of diabetes among patients with pancreatic cancer, and identify the factors associated with type 2 diabetes mellitus in this population.

**Methods:**

This retrospective observational and analytical study was carried out in the Department of Gastroenterology of the Mohammed VI University Hospital over a period of 5 years, between 2018 and 2022, including all patients with confirmed cases of pancreatic adenocarcinoma.

**Results:**

Out of the 197 patients, 38.1% had a history of diabetes, among them, 42.7% had new-onset diabetes, while the remaining 57.3% had long-standing diabetes. Diabetic patients were significantly older than nondiabetic patients (mean age of 67.2 vs. 63, P = 0.009). Diabetes was more prevalent among obese patients (66.7%, P = 0.01), and less frequent among individuals with chronic alcohol consumption (20% vs. 80%, P = 0.04), and tobacco smokers (24.4% vs75.6%, P = 0.03). Among patients with an ECOG score ≥ 3, DM, 54.5% were DM-patients (P = 0.033). The same significant association was found for the Nutritional Risk Index, Patients who had moderate or severe malnutrition were more likely to be diabetic 74.7% (P = 0.004). Diabetic patients were less likely to undergo surgery due to comorbidities and general health deterioration. However, no significant differences were observed in sex, tumor stage or location.

**Conclusion:**

Our study has shown an increased prevalence of diabetes in pancreatic cancer and highlights the importance of considering this cancer in cases of recent onset or uncontrolled diabetes, especially in elderly individuals.

## Background

Pancreatic cancer (PC) is known for its high incidence of advanced-stage disease upon diagnosis and its resistance to treatment, making it a highly aggressive malignancy with an unfavorable prognosis. The predominant type of PC is pancreatic ductal adenocarcinoma (PDAC), which constitutes over 90% of pancreatic tumors [1]. It is the fourth leading cause of cancer death in the United States, and the seventh-leading cause worldwide. By 2030, based on the current rising trends, PC is expected to become the second highest cause of cancer-related mortality in the United States [2]. Some of the primary risk factors associated with pancreatic cancer, although not necessarily exhaustive, include a family history of the disease, genetic mutations, smoking, a sedentary lifestyle, obesity, diabetes mellitus (DM), and chronic pancreatitis.

Individuals with diabetes have a twofold increased risk of developing different types of cancer, including colorectal, endometrial, renal, breast, liver, and pancreatic cancer [1].

Diabetes mellitus (DM) is a chronic and significant medical condition characterized by inadequate production of insulin by the body or the inability to effectively utilize the insulin it produces, its prevalence has reached alarming levels, making it a major health concern. In 2021, the estimated number of individuals affected by diabetes stands at 537 million, and projections indicate that this figure will rise to 643 million by 2030. Type 2 diabetes (T2DM) is the most common form, comprising over 90% of diabetes cases worldwide [3].

Roughly 50% of PC patients exhibit either type 2 diabetes mellitus (T2DM) or impaired glucose tolerance during the early stages. T2DM is a well-established risk factor for PC, and the new-onset diabetes (NOD) could be an early manifestation of PC [4].

The aim of this study is to investigate the prevalence of diabetes in pancreatic cancer in a North African country, as well as certain characteristics and factors associated with diabetes in this deadly disease.

## Methods

We conducted a retrospective study with a descriptive and analytical approach, over a period of 5 years, between January 2018 and December 2022, in the Department of Gastroenterology of the Mohammed VI University Hospital of Oujda (which is considered the reference center for pancreatic cancer diagnosis in the Oriental region of Morocco), in collaboration with the Laboratory of Epidemiology, Clinical Research, and Public Health (LERCSP) of the Faculty of Medicine and Pharmacy of Oujda.

We included:

- All patients of both sexes with confirmed cases of PDAC based on the results of the pathological examination of the tumor sample obtained by endoscopic ultrasound, biopsy of hepatic metastases, brush cytology of the biliary tract or the operative specimen after resection in resectable cancer.
- In our research, we utilized both fasting blood glucose (FBG) levels and hemoglobin A1c (HbA1c) levels to diagnose diabetes in the participants. Consistent with current medical standards and guidelines, a diagnosis of diabetes was established for individuals exhibiting either elevated FBG (≥ 126 mg/dL or 7.0 mmol/L) or elevated HbA1c levels (≥ 6.5%). We ensured that the diagnostic criteria used were consistent and reliable in assessing the prevalence of diabetes in our study population.

We excluded:

- Patients with clinical and radiological suspicion of pancreatic cancer, without anatomopathological confirmation.
- Patients who did not have FPG or HbA1c to confirm DM.

The sample size was calculated using the formula: [5]

$$n = \frac{{{z^2} \times p(1 - p)}}{{{\varepsilon ^2}}}\,\,\,\,\,\,\,\,{\rm{with}}:\varepsilon = 5\% ,\,z = 1,96,\,a = 5\%$$

For each patient, comprehensive data were collected, including demographic information, family history of cancer, personal history of diabetes, smoking and alcohol consumption. Additionally, detailed information was gathered on the date of pancreatic cancer diagnosis, presenting symptoms, laboratory test results, tumor location and size, and cancer stage.

In our study, NODM specifically pertains to patients who were diagnosed with diabetes either concomitantly with the diagnosis of pancreatic cancer or within a defined period, such as 1 years before the diagnosis of pancreatic cancer.

Quantitative data were expressed as the mean ± standard deviation (SD) or median values with range. Qualitative variables were reported as numbers with percentages. Group comparisons used chi2 tests and Student’s t-test for comparing two means. A P value < 0.05 was used to characterize statistically significant results.

The statistical analyses were accomplished by IBM SPSS Statistics version 21.0.

## Results

During the study period, 197 patients met our inclusion criteria. The mean age of our patients was 64.6 ± 11.5, ranging from 33 to 91 years and 90.9% were aged over 50. Men represented 50.3%. of the cases studied (n = 99), while women constituted 49.7%) (n = 98), with an M/F sex ratio of 1.01.

The prevalence of diabetes in our study population was 38.1% with a 95% confidence interval [31.5–45.0]. Among them, 42.7% had been diagnosed with new-onset diabetes (NODM) within a year prior to their cancer diagnosis, while the remaining 57.3% had long standing diabetes with an average duration of 9 +/- 6.1 years before diagnosis (ranging from 2 to 25 years). The mean age of patients with NODM was higher than those with long standing-DM (62 years vs. 55 years, P = 0.04). Thirty- two% of DM-patients were taking insulin, while 68% were taking oral hypoglycemic agents. At the time of diagnosis, 90% of the DM-patients had uncontrolled diabetes (hyperglycemia and elevation of HbA1c).

A history of familial pancreatic cancer was found in 3% of cases, obesity in 7.6%, chronic tobacco smoking in 22.8%, and chronic alcohol consumption in 12.7%.

Symptoms preceding PC diagnosis included abdominal pain (88.8%), jaundice (56.3%), vomiting (28.4%), weight loss (94.9%), constipation (7.1%), and diarrhea (3%). Out of the total participants, 16.7% had an ECOG performance scale score of 3 or more, and there was a significant association between ECOG score and cancer stage, with 65% of metastatic cancers having a score of 3 or more (p = 0.02).

The imaging data revealed that head cancer represented 80.7% of all cases, while corporal and caudal locations accounted for 19.3%. Among the cases, 19.8% of cancers were resectable, 4.1% were borderline, 43.7% were locally advanced, and 32.4% were metastatic. (Table 1)

**Table 1:** Summary of the demographic and baseline characteristics and radiological findings of the patients :

Variable	Number	Percent %
**Age**: mean & SD:	64.6 ± 11.5	
**Sex** Female Male	98 99	49.9 50.3
**Tabacco smoking** No Yes	152 45	77.2 22.8
**Alcohol consumption**		
No Yes	172 25	87.3 12.7
**Diabetes** No Yes	122 75	61.9 38.1
**Obesity** No Yes	182 15	92.4 7.6
**Familial history of pancreatic cancer** No Yes	191 6	97 3
**Abdominal pain** No Yes	22 175	11.2 88.8
**Jaundice** No Yes	86 111	43.7 56.3
**Weight loss** No Yes	97 100	49.2 50.8
**Vomiting** No Yes	141 56	71.6 28.4
**ECOG ≥ 3** No Yes	164 33	83,3 16.7
**Site** Head Body and tail	159 38	80.7 19.3
**Stage** Resectable Borderline Locally advanced Metastatic	39 8 86 64	19.8 4.1 43.7 32.4

The comparison between DM and non-DM patients did not reveal a significant difference in terms of sex. However, we found a statistically significant association between age and diabetes. DM-patients had a higher mean age than non-DM individuals (67.2 vs. 63 years, P = 0.009). Dyslipidemia was found to be significantly associated with diabetes, and 90.9% of patients with dyslipidemia had DM (p < 0.001). Obesity was also found to be associated with diabetes, 66.7% of obese patients had diabetes (P = 0.01). Among patients with chronic alcohol consumption, there was a higher proportion of non-DM (80%, P = 0.04), and chronic tobacco smoking (75.6%, P = 0.03). Regarding the clinical signs, the frequency of diabetes was higher in patients who had vomiting (53.6%, P = 0.005), while there was no significant difference between DM and non-DM patients for abdominal pain, jaundice, diarrhea, and constipation.

A significant association was found between the ECOG score and diabetes. Among patients with an ECOG score ≥ 3, 54.5% were DM patients (P = 0.033). The same significant association was found for the Nutritional Risk Index, patients who had moderate or severe malnutrition were 74.7% more likely to be diabetic (P = 0.004). A carbohydrate antigen 19 − 9 (CA 19 − 9) level higher than 100 was more frequent among non-DM patients (55.3%, P = 0.006). Regarding the imaging features, there was no significant association between DM and size, location, or cancer stage.

In multivariate analysis, only Age, Obesity, and CA19-9 were found to be associated with diabetes in patients with pancreatic cancer, with a p-values of 0.022 for Age, 0.019 for Obesity, and 0.012 for CA19-9 (Table 2).

**Table 2:** Binary logistic regression between DM and the explanatory variables

	OR (95% IC)	P-value
Age	1.032(1.005–1.060)	0.022
Obesity No Yes	1 4.150(1.262–13.645)	0.019
CA 19 − 9 ≤ 100 ml > 100 ml	1 2,394(1,212-4,727)	0.012

For resectable cancers, surgery was performed in 75.9% of non-DM patients and only in 30% of DM-patients (P = 0.009). This was attributed to comorbidities, malnutrition, and general health deterioration among DM-patients. (Table 3)

**Table 3:** Univariate Analysis of the Association between Diabetes and Demographic, Baseline Characteristics, and Laboratory/Radiological Findings in Patients with Pancreatic Cancer

	DM 75(38.1%)	Non-DM 122(61.9%)	P value
**Mean age**	67 ± 10	63 ± 12	0.009
**Gender** Men Women	33 (33.3) 42 (42.9)	66 (66.7) 56 (57.1)	0.169
**Chronic tobacco Smoking**	11 (24.4)	34 (75.6)	0.032
**Alcohol consumption**	5 (20)	20 (80)	0.046
**Obesity**	10 (66.7)	5 (33.3)	0.018
**Jaundice**	45 (40.5)	66 (59.5)	0.41
**Abdominal pain**	68 (38.9)	107 (61.1)	0.522
**Vomiting**	30 (53.6)	26 (46.4)	0.005
**Weight loss**	75 (40.1)	112 (59.9)	0.001
**ECOG ≥ 3**	18 (54.5)	15 (45.5)	0.033
**Tumor size (< 2 cm)**	10 (25%)	30 (75)	0.057
**Site** Head Body and tail	62 (39) 13 (34.2)	97 (61) 25 (65.8)	0.585
**Tumor stage** Resectable Unresectable Metastatic	10 (25.6) 38 (40.4) 27 (42.2)	29 (74.4) 56 (59.6) 37 (57.8)	0.198
**CA 19 − 9 > 100 UI/mL**	16 (44.7)	59 (55.3)	0.006
**Operable patients among resectable cancers**	3 (30%)	22 (75.9%)	0.009

## Discussion

Our study revealed that the prevalence of diabetes among patients with pancreatic cancer was 38.1%: 42.7% had a NODM, while the remaining 57.3% had long standing diabetes, and 90% of them had uncontrolled diabetes. Also, DM-patients were older than non-DM individuals (mean age: 67.2 vs. 63 years, P = 0.009). This finding underscores the importance of considering pancreatic cancer in individuals with recent or uncontrolled diabetes, especially in the elderly.

The observed prevalence is consistent with many recent studies that have explored the complex and bidirectional relationship between PC and type 2 DM (T2DM). Long-standing T2DM represents a notable risk factor for various malignancies, including PC. Several studies have indicated that approximately 85% of patients diagnosed with PC had also concurrent diabetes at the time of diagnosis [6]. Patients with long-standing T2DM (≥ 5 years) have a 50% increased risk of PC compared with non-DM patients [7].

Obesity was associated to diabetes in our PC population, with 66.7% of obese patients having diabetes (P = 0.01). On the other hand, chronic alcohol consumption (P = 0.04) and chronic tobacco smoking (P = 0.03) were less prevalent among patients with DM. Studies exploring the role of metabolic alterations related to obesity has supported the risk and indicated that elevated insulin levels resulting from obesity-related insulin play a vital factor. These findings are consistent with previous studies and highlight the impact of lifestyle factors on the risk of diabetes in PC. Stolzenberg-Solomon et al. conducted a study on 29,133 Finnish male, and they found that higher fasting glucose, insulin levels, and insulin resistance were positively linked to PDAC, Given that obesity is associated with insulin resistance in almost all subjects, hyperinsulinemia is believed to play a causative role in the development of PDAC [8].

According to the mechanism of the association between diabetes and PC, it’s hypothesized that high levels of insulin, particularly within the pancreas itself, resulting from obesity, insulin resistance in prediabetes, or T2DM, could lead to proxicrine effects on nearby acinar and ductal cells, thereby promoting their survival and proliferation. The Sustained hyperinsulinemia and the continuous impetus by beta cells to overcome insulin resistance and maintain glucose homeostasis may play a role in this observed association [9].

Conversely, it has been observed that DM can be a result of PC. In a previous study, it was found that approximately 57% of patients with pancreatic cancer and NODM experienced the resolution of their diabetes following pancreaticoduodenectomy. These findings strongly suggest that NODM in the context of pancreatic cancer is likely a result of the tumor itself [10]. The association between NODM and pancreatic cancer is not well understood. Among the hypotheses mentioned, the tumor itself may produce substances that increase resistance to insulin in muscle and adipose tissues. Surgical removal of small tumors, while preserving the insulin-producing islets in the tail of the pancreas, can improve insulin sensitivity and effectively eliminate diabetes, further supporting this hypothesis. Additionally, in PDAC, there is evidence of islet blood flow dysfunction, microthrombosis, and perivascular fibrosis. These factors likely contribute to the suppression of normal insulin secretory dynamics in individuals with NODM [11].

There are a few studies that compare NODM and long-standing DM in PC. In a large longitudinal study, the risk of PC was found to be more important in patients with NODM (3 years or less) compared to individuals with DM for persisting for more than 3 years, with a hazard ratio (HR) of 1.55 [12].

Regarding T1DM, its association with PDAC is not clearly established, because of the limited number of patients with T1DM compared to those with T2DM [11]. A large cohort study conducted in Sweden (29,187 individuals with T1DM) found no statistically significant increase in the risks of PC in this population. These findings support the theory that chronic endogenous hyperinsulinemia plays a role in the higher occurrence of PDAC in patients with longstanding T2DM [13].

In our context, DM was associated with specific clinical symptoms in PC patients. Vomiting was more frequent among diabetic patients (53.6%, P = 0.005), while no significant differences were observed for other symptoms. Additionally, there was no significant association between diabetes and tumor stage, suggesting that diabetes may not influence the extent of PC at the time of diagnosis.

Our finding align with a systematic review that examined the impact of DM on clinical outcomes of PC found that DM-patients had a higher prevalence of male sex (odds ratio [OR] 0.81, 95% confidence interval [CI] 0.69–0.95; P = 0.01) and a higher body mass index (weighted mean difference [WMD] 1.45, 95% CI 0.60–2.30; P < 0.001). However, there were no significant differences observed in terms of age, smoking history, and the presence of jaundice, tumor location, and cancer stage between the two groups. Diabetic patients did not have a higher risk for overall morbidity or pancreatic fistula [14]. Similarly, *Toriola et al.* reported comparable characteristics in patients with and without DM, except for BMI. However, median survival was shorter for individuals with diabetes (92 days) than for those without diabetes (139 days), (p value of 0.05) [15].

On the other hand, a large population-based cohort study conducted by Allen Hwang et al., individuals with T2DM were found to be significantly older, more likely to be male, and had a higher prevalence of pancreatic resection, smoking history, higher Charlson index, and higher BMI. However, the study did not find a significant difference in overall survival at the time of PC diagnosis. Interestingly, when considering different durations of T2DM, it was found that individuals with a preexisting T2DM duration of over 5 years had an increased mortality rate, compared to NODM [16].

Contrary to our study, a case-control study conducted at MD Anderson Cancer Center, patients with DM had a larger tumor size (> 2 cm), and elevated levels of CA19-9. DM was also associated with a decrease of 1.2 months in overall survival (OS) time for all patients (P = 0.041). In patients who underwent tumor resection, the association was even more significant, with a reduction of 11 months in OS (P = 0.025) [17].

It is crucial to acknowledge the limitations of our study: the retrospective nature of our study might introduce some biases. Additionally, post-resection changes in DM parameters were not examined in this study. Therefore, a longitudinal study can provide more data for analysis, focusing on assessing the impact of pancreatic cancer treatment on diabetes management and its relation to prognosis.

## Conclusion

Our study revealed a high prevalence of diabetes in patients with pancreatic cancer, with a substantial proportion of cases being new-onset diabetes. This highlights the need for increased awareness and consideration of pancreatic cancer in individuals with recent or uncontrolled diabetes, especially in the elderly. The study also identified several factors associated with diabetes in this population, including age, obesity, chronic alcohol consumption, and tobacco smoking. Further research is required to understand the underlying mechanisms and explore potential interventions, like longitudinal studies that follow individuals with diabetes over time and investigate the impact of diabetes duration on pancreatic cancer risk and prognosis could provide valuable insights.

## Data Availability

The datasets used and analyzed during the current study are available from the corresponding author on reasonable request.
